# Stroke Prevention and Treatment for Youth with Sickle Cell Anemia: Current Practice and Challenges and Promises for the Future

**DOI:** 10.1007/s11910-024-01372-9

**Published:** 2024-09-21

**Authors:** Susan Creary, Melissa G. Chung, Anthony D. Villella, Warren D. Lo

**Affiliations:** 1 Division of Hematology/Oncology/BMT, Dept of Pediatrics, The Ohio State University and Nationwide Children’s Hospital, 700 Children’s Drive, Columbus, OH 43205 USA; 2 Division of Neurology, Dept of Pediatrics, The Ohio State University and Nationwide Children’s Hospital, 700 Children’s Drive, Columbus, OH 43205 USA; 3 Division of Critical Care, Dept of Pediatrics, The Ohio State University and Nationwide Children’s Hospital, Columbus, OH 43205 USA

**Keywords:** Stroke, Sickle cell anemia, Prevention

## Abstract

**Purpose of Review:**

Sickle cell anemia (SCA) is an autosomal recessive inherited hemoglobinopathy that results in a high risk of stroke. SCA primarily affects an underserved minority population of children who are frequently not receiving effective, multi-disciplinary, preventative care. This article reviews primary and secondary stroke prevention and treatment for children with SCA for the general adult and pediatric neurologist, who may play an important role in providing critical neurologic evaluation and care to these children.

**Recent Findings:**

Primary stroke prevention is efficacious at reducing ischemic stroke risk, but it is not consistently implemented into clinical practice in the United States, resulting in these children remaining at high risk. Acute symptomatic stroke management requires neurology involvement and emergent transfusion to limit ischemia. Furthermore, while chronic transfusion therapy is a proven secondary preventative modality for those with prior symptomatic or silent cerebral infarcts, it carries significant burden. Newer therapies (e.g., stem cell therapies and voxelotor) deserve further study as they may hold promise in reducing stroke risk and treatment burden.

**Summary:**

Effective primary and secondary stroke prevention and treatment remain a challenge. Informing and engaging neurology providers to recognize and provide critical neurologic evaluation and treatment has potential to close care gaps.

## Introduction

### Sickle Cell Anemia, Stroke, and Neurological Concerns

Pediatric arterial ischemic stroke after the neonatal period is an uncommon disorder, affecting 1–2/100,000 children per year in Western developed countries [1] Primary prevention for childhood arterial ischemic stroke is challenging because of the low frequency and because modifiable risk factors occur much less commonly than those for adult stroke. One exception is children who have SCA. Because their risk factors are known, primary and secondary prevention are feasible, and the benefits of intervention can be measured.

While there have been earlier reviews of stroke prevention in patients with SCA, [2–5] these were not targeted to the general practicing neurologist. This review focuses upon the identification and treatment, primary prevention, and secondary prevention of stroke in youth with SCA. Many of the current SCA stroke prevention options and related treatments may not be familiar to general adult and child neurologists in North America who do not work in a tertiary sickle cell disease center. Yet these neurologists may provide care to children and young adults, especially given the gaps in access to care and treatment that are common to this population. This review will identify instances where patients with SCA may not receive the recommended primary stroke prevention screening measures. It will discuss the challenges and barriers to implementing secondary stroke prevention treatment and the potential role for newer therapies in the management of stroke in SCA. Lastly this review will touch upon the gaps in our knowledge for stroke prevention and management in young adults with SCA.

#### Sickle Cell Anemia and Stroke

SCA includes individuals with Hemoglobin (Hb) SS and HbSβ^0^thalassemia and is the most common and severe form of sickle cell disease, a hemoglobinopathy resulting in erythrocyte sickling. This ultimately leads to tissue ischemia, inflammation, and vasculopathy that can lead to pain, stroke, and numerous debilitating complications beginning in early childhood. While SCA can affect people of all races, it is more commonly found in people whose ancestors came from Sub-Saharan Africa, parts of the Mediterranean, the Middle East, and India [6]. In the US, it affects 100,000 Americans [7] many of whom are underserved minorities who frequently do not receive multi-disciplinary preventative care [8].

SCA increases stroke risk > 200 fold, [9] making SCA a large contributor to overall stroke incidence in childhood [10]. The mechanisms of stroke in SCA are complex and likely a result of anemia, abnormal properties of sickled erythrocytes, altered blood flow, and interactions with the endothelium [11]. Individuals with SCA are at risk for different types of strokes that can co-occur in the same individual. The field changed with the landmark STOP trial, (Table 1) [12] which selected children with SCA who had no history of stroke, but who were at high risk based upon transcranial Doppler studies of the internal carotid or middle cerebral arteries. This trial showed that chronic transfusion that maintained the HbS% below 30% significantly reduced the risk of first stroke.

**Table 1:** Key observational study and clinical treatment trials of children with SCA, and the impacts of these studies upon clinical practice

Trial	Population*	Intervention (vs Comparison)	Outcome (and Timeline)	Key Findings
The use of transcranial Doppler ultrasonography to predict stroke in sickle cell disease [13]	≥ 3 years without a prior history of stroke	Biannual transcranial Doppler screening until age 21 years	Overt cerebral infarction	Transcranial Doppler screening can identify children with SCA who are at highest risk of arterial ischemic stroke
STOP 1[12]	2–15 years of age, without prior history of stroke but elevated transcranial Doppler screen	Chronic transfusions vs no disease modifying treatment	Cerebral infarction or intracranial hemorrhage	Chronic transfusions can significantly reduce the risk of stroke in children with SCA identified as high risk by transcranial Doppler screening
STOP 2 [14]	2–15 years of age, elevated transcranial Doppler screen that normalized, who had received ≥ 30 months of chronic transfusions	Chronic transfusions vs no disease modifying treatment	Composite endpoint of cerebral infarction or intracranial hemorrhage or reversion to elevated transcranial Doppler screen	Stopping chronic transfusions results in a high rate of reversion to elevated transcranial Doppler screen and stroke
SWITCH [15]	5–19 years who had a prior symptomatic ischemic stroke and had received at least 1.5 years of chronic transfusion	Hydroxyurea and phlebotomy vs. chronic transfusions and iron chelation	Composite endpoint of secondary stroke recurrence and liver iron	Ongoing chronic transfusions and iron chelation are better than hydroxyurea and phlebotomy to prevent secondary stroke and iron overload
TWITCH [16]	4–16 years who had an abnormal transcranial Doppler screen, no severe vasculopathy, and had received at least 1 year of chronic transfusions	Hydroxyurea vs. chronic transfusions	Transcranial Doppler velocity at 24 months	After a year of chronic transfusions, hydroxyurea is a reasonable alternative to transfusions to prevent primary stroke in children with SCA who are at high risk of stroke and who do not have vasculopathy
SIT [17]	5–15 years of age with no history of arterial ischemic stroke but ≥ 1 silent cerebral infarct (SCI) on magnetic resonance imaging	Chronic transfusions vs no disease modifying treatment	New overt stroke or new or enlarged SCI	Chronic transfusions reduce the infarct recurrence in those with SCI, but the number needed to treat with chronic transfusions to prevent one new infarct was 13 patients for three years

*studies included only children with SCA

In the era after the STOP trial, the incidence of acute ischemic stroke in children with SCA is 0.24 per 100 patient-years [18]. Silent cerebral infarcts (SCI), or ischemic injuries that are not associated with acute neurological deficits but detected on magnetic resonance imaging (MRI), are much more common. SCI occur in approximately one fourth of children by age 6 [19], one third by age 14, and have devastating impacts on cognitive functioning and school achievement [20]. SCI in children increase the risk of arterial ischemic stroke and subsequent SCI [21]. Finally, SCA can result in severe vasculopathy, i.e. moyamoya syndrome, that results in a high risk of ischemic and hemorrhagic stroke despite intervention [22, 23].

In high-resource countries such as the U.S. and Canada, SCA is identified by universal newborn screening prior to the appearance of clinical complications. Early identification allows for initiation of prophylactic antibiotics to prevent life-threatening encapsulated bacterial infections [24] and referral for tertiary, multi-disciplinary care to prevent disease complications including stroke. In low-resource settings such as sub-Saharan Africa, Latin America, the Caribbean, India, the Middle East, and Asia, newborn screening for SCA is uncommon [25]. In those regions most children are diagnosed when overt SCA symptoms manifest. Therefore, SCA must remain on the differential diagnosis for a variety of neurological presentations, since children may have been born in low-resource settings and immigrated to higher-resource settings but may not yet have been identified as having SCA. Unfortunately, even for children born in North America, gaps in care suggest that more than half of children with SCA do not receive routine primary stroke prevention measures [26, 27]. Thus, general neurologists should be aware how children with SCA may present with neurological symptoms and be aware of the prevention and treatment modalities that are available to children with SCA.

### Opportunities for Primary Acute Ischemic Stroke Prevention in SCA

#### High Risk Patient Identification

While primary prevention for arterial ischemic stroke in older adults includes controlling the multiple risk factors that contribute to atherosclerosis, primary prevention in children with SCA is based upon identifying which of these children are at high risk for developing cerebral ischemia. This is achieved by annual screening of children from age 2–16 years with transcranial Doppler ultrasound to measure the blood flow velocities in the distal internal carotid and proximal middle cerebral arteries. Transcranial Doppler measurement should not be performed soon after a transfusion, as there is an association between recent transfusion and transient normalization of transcranial Doppler velocities [28].

Normal time averaged mean maximum velocities (TAMMV) in these vessels are 70 to < 170 cms/second using non-imaging duplex devices when children are at their baseline state of health. If TAMMV in either of these vessels is between 170 to ≤ 200 cms/sec, the velocity is considered “conditional” and more frequent screening is recommended (e.g., every 3 months). However, if TAMMV in one or more of these vessels is ≥ 200 cms/sec on repeated measurements performed over 2–4 weeks, this is defined as abnormal. Children with abnormal transcranial Doppler screens should be treated [29] because clinical trial data shows that they have a 44-fold relative risk of stroke [13].

Despite data suggesting that inadequate screening is a significant contributor to stroke occurrence [30–32] and subsequent analyses indicating a higher risk of ischemic stroke in children who did not have transcranial Doppler screenings or chronic transfusions performed according to protocol [18], less than 50% of eligible children with SCA living in the United Sates receive annual transcranial Doppler screenings, and this low rate of screening has remained unchanged for decades [27, 33]. Barriers to screening include poor access to care, insufficient numbers of providers who can perform and interpret transcranial Doppler studies, [34] significant inconsistencies with TAMMV performance, [35] and poor understanding by patients and their caregivers about the need for and importance of regular screening [36]. The persistence of these gaps suggests that neurology providers must remain aware that new neurological signs and symptoms in children with a known diagnosis of SCA may reflect a new ischemic stroke that was not anticipated due to inadequate screening.

#### Primary Preventative Treatment

Chronic transfusions are recommended for primary prevention in high-risk patients with SCA because chronic transfusions significantly reduce the risk of symptomatic ischemic stroke. (STOP trial) (Table 1) A subsequent trial (STOP2) [14] showed that stopping chronic transfusion without initiating any other treatment quickly led to a return of abnormal transcranial Doppler velocities and an increased risk for ischemic strokes. Thus, in high-resource settings, guidelines recommend that children with SCA and abnormal transcranial Doppler screens receive chronic transfusions to achieve a HbS% of < 30% and a total hemoglobin concentration > 9 g/dL for at least 12 months to reduce stroke risk [12] (STOP trial) (Table 1). The findings from Adams et al. 1992 and the STOP trials were used to support the most recent published guidelines that recommend annual transcranial Doppler screening for children who have SCA and other genotypes with hemolysis characteristics similar to those with SCA [29].

Because long-term chronic transfusion has many disadvantages (e.g., chronic iron overload, alloimmunization [37]. intravenous access challenges, and the burden of attending monthly transfusions) [38] the TWITCH trial (Table 1) explored hydroxyurea as an alternative to chronic transfusions for primary stroke prevention. It showed that if patients do not have silent cerebral infarcts (SCI) or severe vasculopathy such as moyamoya syndrome by MRI and MRA, bridging to hydroxyurea [16] after one year of chronic transfusions is an option [29]. Furthermore, emerging data indicates that in patients who undergo hematopoietic stem cell transplantation, cerebral blood flow and cerebrovascular stenosis may normalize 2–3 years post-transplant [39, 40].

### Case 1

12-year-old male presented to the emergency department with acute left sided weakness of arm and face with no difficulty talking, or vision changes. There was no history of prior similar episodes. He had no recent headache, infections, trauma. Past medical history was significant for attention problems. Family history was significant for a maternal grandmother who had a stroke in her 50 s, and a maternal history of sickle cell trait. Paternal family history was unknown. Social history was significant for immigration from West Africa at age 8 and poor school performance.

Significant neurological exam findings were a left facial droop with intact forehead movement, leftward tongue deviation, inability to move left upper and lower extremities except to painful stimuli. The remainder of his neurological exam including language and ability to answer questions was normal. He had a 2 + flow murmur but otherwise his physical exam was normal.

**Fig. 1.**
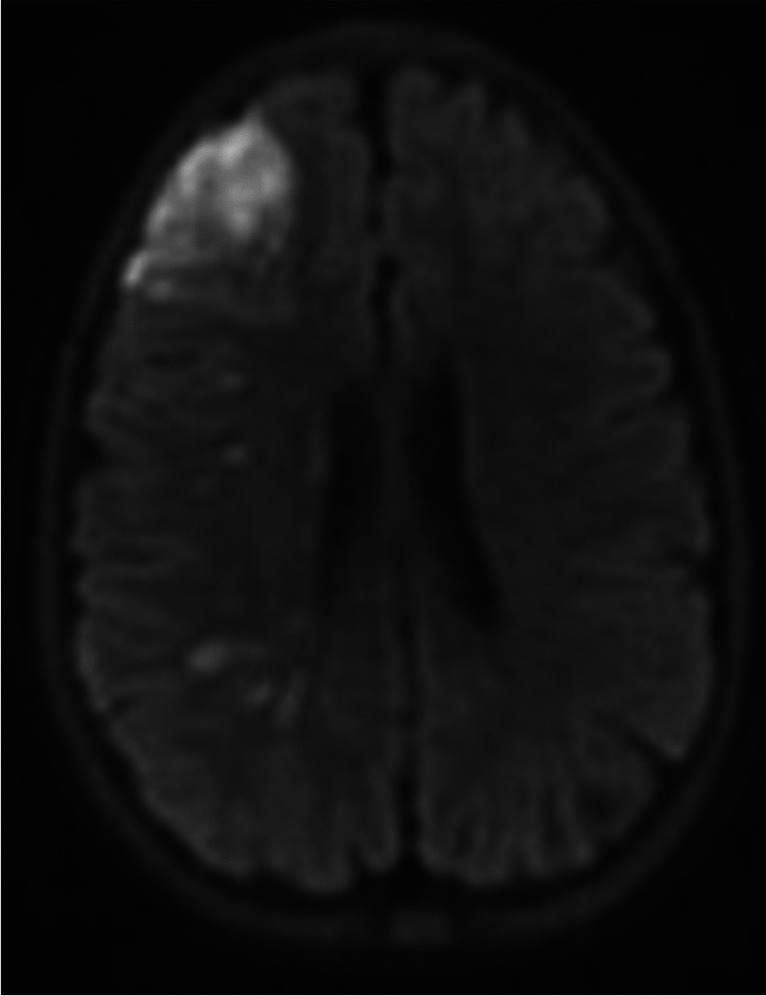
Acute diffusion changes in the right middle cerebral artery distribution consistent with a right MCA distribution infarct

Laboratory studies were notable for: Hemoglobin 7.3 g/dL, platelet count 462 K/cu mm, reticulocyte count > 20%. MRI showed an acute right middle cerebral artery infarction (Fig. 1). Given the history, laboratory findings of a severe anemia, and imaging findings, acute ischemic stroke related to suspected SCA was considered and the patient was admitted to the intensive care unit to receive an emergent exchange transfusion.

This case illustrates one clinical setting when one should have a heightened level of suspicion for SCA-related complications. Not all children with SCA may be identified, particularly those immigrating from countries without newborn screening. Arterial ischemic stroke may be the first symptomatic manifestation of SCA, which must be on the differential diagnosis for acute or chronic arterial ischemic stroke for children born in global regions where SCA occurs more frequently [6]. Even children known to have SCA may not have had adequate primary prevention screening or treatment, so if they present with an acute or sub-acute neurological deficit consistent with a cerebrovascular event, an MRI to detect cerebral ischemia and empiric treatment should be pursued.

## Acute Treatment of Arterial Ischemic Stroke in Patients with SCA

While a detailed review of acute stroke management is beyond the scope of this review, there are important differences in the acute management of stroke in those with SCA, especially for children. If a child with SCA presents with primarily small vessel disease, this likely is mediated by the amount of sickled cells in their circulation. A critical step in treatment is the reduction of HbS% to < 15% in the circulation. This is most efficiently and effectively performed by exchange transfusion, but this requires specialized resources that may not be available in smaller community hospitals. In that case and for children with significant anemia (e.g., Hb < 9 g/dL) HbS% can be acutely lowered with a simple transfusion, while efforts to arrange exchange transfusion and/or transfer to a center that can perform this are under way. Also, children with SCA typically do not have the risk factors for stroke that are seen in adults. Therefore, current guidelines recommend against intravenous tissue plasminogen activator in children with SCA. In young adults with SCA, intravenous thrombolysis is a consideration, particularly if additional risk factors for stroke are present, but transfusion is still a key element for acute management [29].

While endovascular thrombectomy has opened up considerable opportunities for the treatment of ischemic strokes due to large vessels occlusions, [41, 42] endovascular thrombectomy in children and young adults with SCA should be pursued cautiously given the association of moyamoya syndrome and SCA. SCA related moyamoya vasculopathy can involve large vessels such as the internal carotid artery, middle cerebral artery, or anterior cerebral arteries, and the M1 branch of the middle cerebral artery. Cannulation of a moyamoya stenotic vessel may risk a perforation or dissection, yet these moyamoya-associated large vessel occlusions may be difficult to distinguish from large vessel occlusions due to another cause. The presence of multiple lenticulostriate or thalamostriate collaterals may serve as additional warning that moyamoya syndrome may be present and if endovascular thrombectomy is considered in patients with SCA, the interventionalist should be made aware that the patient has SCA and that there is a possibility of moyamoya vasculopathy [43].

## Secondary Stroke Prevention

### Arterial Ischemic Stroke

Children who suffer an arterial ischemic stroke have a long-term elevated risk for recurrent ischemic stroke. Current guidelines recommend that these individuals remain indefinitely on chronic transfusions for secondary stroke prevention [29]. Alternatively, hematopoietic stem cell transplantation (HSCT) is curative and a definitive option for secondary stroke prevention in those children who are fortunate to have matched siblings or haploidentical donors [44, 45]. Donor availability and the risks associated with HSCT limit the greater use of HSCT. Long-term risks and complications of chronic transfusions may limit some patients from continuing chronic transfusions indefinitely (i.e., inadequate intravenous access, alloimmunization), thus some may require switching to alternative therapies, such as hydroxyurea, that are unproven at preventing subsequent stroke.

### Silent Cerebral Infarcts (SCI)

SCIs are much more common than arterial ischemic stroke, but the mechanisms that produce SCI are less understood, and prevention is problematic. The long-term negative impacts of SCI are significant and include an increased risk for arterial ischemic stroke [21] and cognitive and executive functioning deficits [20, 46]. Current evidence indicates that SCI occur more often in regions with low cerebral blood flow, such as the deep white matter [47]. A randomized trial showed that chronic transfusions reduce the frequency of subsequent SCI but do not completely prevent subsequent SCI or arterial ischemic stroke [17]. Recent data suggests that haploidentical transplantation may improve cerebral blood flow dynamics in adults [48]. While this may present an alternative to chronic transfusions for SCI prevention, [49] studies that test if alternative treatments such as hydroxyurea, HSCT, or gene therapy are effective at preventing subsequent SCI have not yet been completed. Thus, lifelong chronic transfusions for children with SCI may need to be considered, despite encountering the same long-term risks associated with using chronic transfusions for secondary arterial ischemic stroke prevention.

Because SCIs may not be associated with focal neurological signs, current guidelines advise that providers should perform developmental and cognitive surveillance and at least one MRI brain scan during childhood to look for SCI. [29] Developmental and cognitive surveillance includes screening for developmental delays in preschool-aged children and academic or behavioral problems or symptoms of inattention, hyperactivity, or impulsivity in older children. While conditions such as attention-deficit hyperactivity disorder may be present in children with SCA, children flagged by developmental and cognitive screening should have a formalized evaluation of behavior and cognitive functions by psychologists or neuropsychologists, and MRI brain imaging to evaluate for underlying SCI.

## Moyamoya Syndrome/Vasculopathy

Moyamoya syndrome is a severe complication of SCA marked by progressive stenosis and occlusion of the large cerebral vessels. Typically, the carotid or middle cerebral arteries are affected although the anterior and posterior cerebral arteries may also be involved. The pathophysiology contributing to the development of moyamoya syndrome in SCA is poorly understood, [50] although hemoglobin polymerization, chronic hemolysis, release of free hemoglobin into the circulation, and activation of pro-inflammatory mediators likely contribute [51]. Concurrent with the occlusion of the large cerebral arteries, small collateral lenticulo- and thalamostriate vessels often develop. Moyamoya syndrome may develop gradually and present with nothing more evident than progressively worsening headaches. The vasculopathy may not be identified until the patient presents with a hemorrhagic or ischemic stroke or transient ischemic attack (TIA). Occasionally, though, moyamoya syndrome may be identified in an asymptomatic patient who has an MRI and MRA because of either a significantly elevated (≥ 200 cm/sec) or decreased (< 50 cm/sec) TAMMV on transcranial ultrasound.

Because moyamoya syndrome increases the risk for multiple types of strokes, a number of surgical procedures have been proposed that collectively can be described as revascularization surgeries. Multiple retrospective studies [23, 52–54] have reported a decreased rate of acute strokes, TIA, and SCI in patients with SCA and moyamoya syndrome who had revascularization surgery in addition to medical management. Acknowledging the limitations of this evidence and that there are no trials that directly compare chronic transfusions alone with chronic transfusions and surgical revascularization, current guidelines recommend that those with moyamoya syndrome and a history of stroke or TIA be evaluated for revascularization surgery, in addition to continuing chronic transfusion to prevent the progression of the steno-occlusive process [29]. The optimal medical management of moyamoya syndrome is uncertain. Antiplatelet medications such as aspirin or clopidgrel are frequently used as anti-thrombotic agents although there is no trial-based evidence that supports their use. A small number of studies have shown that HSCT was associated with a) decreased transcranial Doppler velocities more than chronic transfusion [55]; b) a stabilization of the progression of cerebrovascular disease [56]; and c) an improvement in cerebral blood flow and oxygen extraction [40]. This raises the possibility of HSCT as an alternative treatment for moyamoya syndrome that should be explored further. The role of gene therapy remains unknown, as gene therapy trials have included only a few patients with a history of stroke [57, 58].

### Case 2

13 yo female with HbSS received hydroxyurea for 4 years to prevent pain crises; she had normal annual transcranial Doppler exams. At a routine hematology visit her caregiver reported new behavior problems (e.g., getting into fights). There were new academic concerns with a significant decline in school performance. Her grades used to be mostly B’s but had declined to D’s and F’s and she had more difficulty understanding academic subjects.

Physical exam was notable for a flow murmur but was otherwise normal.

Her hemoglobin was 7.7 g/dL, reticulocyte count was 17.8%, but otherwise she had a normal blood count and comprehensive metabolic panel.

**Fig. 2.**
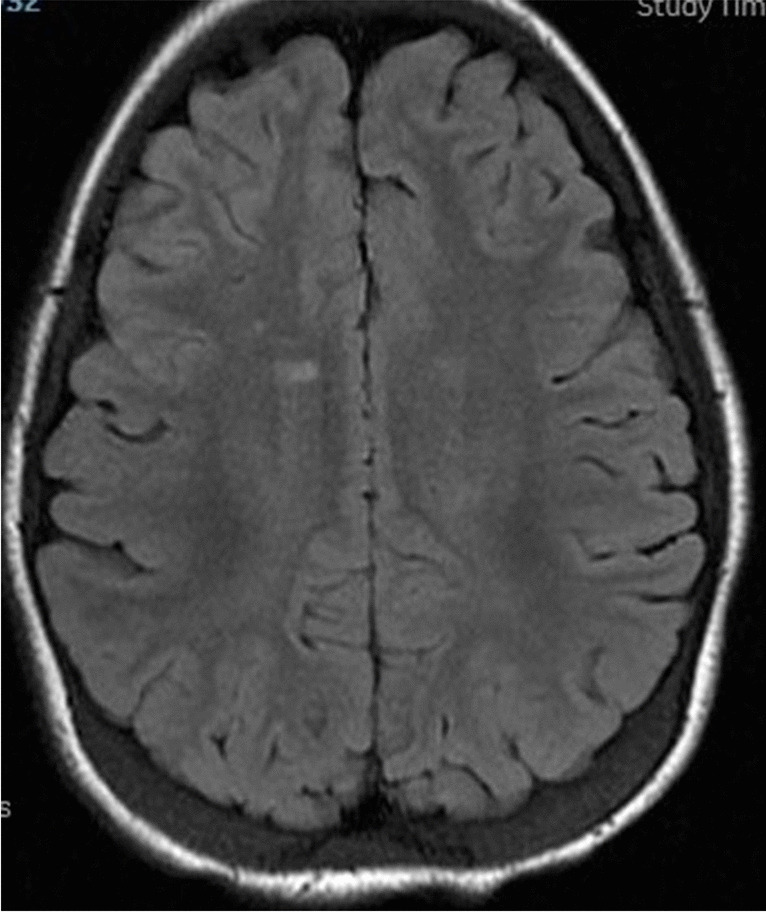
Illustration of T2/FLAIR hyperintensities in the right frontal white matter. The smaller hyperintensity was considered non-specific, but the larger T2/FLAIR hyperintensity was considered an SCI

A non-contrast MRI brain scan and MRA showed several small punctate foci of increased T2 and FLAIR signal in the frontal periventricular white matter but no areas of vascular irregularity, malformation, or occlusion. Otherwise, there was an intact cortical ribbon, normal ventricles, normal basal ganglia, brainstem, and posterior fossa, and a small right choroid fissure cyst. (Fig. 2) Based upon the T2 hyperintensities, her hydroxyurea was discontinued and she was treated with chronic transfusions to prevent subsequent SCIs.

This child illustrates the need for a heightened level of suspicion for SCA-related cerebrovascular complications when a child with SCA is referred for behavior concerns or declining cognitive function. This child also illustrates that despite the use of hydroxyurea, a primary treatment for SCA, SCI may still occur [59]. The presentation can be insidious rather than acute, with behavior changes, academic decline, cognitive deterioration, or attention difficulties, and yet have no focal neurological deficits. Based upon the cognitive and behavior changes, an MRI was obtained in this child, it led to the diagnosis of SCI, and a major change in treatment was the result.

## SCA and Stroke Risk in Young Adults

The concerns with stroke risk and the need for monitoring for the development of SCI continue into young adulthood. The management of young adults with SCA becomes more complicated as these individuals transition from a pediatric system designed to support children and adolescents with SCA to the adult care system [60]. There is limited data regarding the longitudinal outcomes in young adults with SCA. Data from Kassim et al. [61] showed that 9 of 69 (13%) young adults with SCA who had a MRI had symptomatic strokes. After removing the 9 stroke patients from the analysis, of the remaining 60 patients 32 (46%) had at least one silent infarct, and of 55 of the 60 who had MRAs performed, 5 (9%) had saccular aneurysms < 5 mm in size. None of the patients in this series had moyamoya vasculopathy, but of the 9 who were excluded from the analysis because of known strokes 5 had moyamoya vasculopathy. In a follow-up study of 54 of these patients, no new symptomatic strokes occurred, but of the patients who had pre-existing SCI 30% developed new or progressive SCI. In contrast only 6% of patients who did not have a pre-existing SCI developed an SCI. The use of hydroxyurea, the dose of hydroxyurea, and the presence of other stroke factors had no significant effect upon the development of SCI [62]. A third two-site study reported that 50% of adults (median age 26 yrs) had SCI [63]. Together, these three studies show that young adults with SCA have a high risk for stroke and SCI despite available treatment.

Based upon these findings, current guidelines recommend that adults with HbSS or HbSβ^0^ thalassemia have at least one MRI to screen for SCIs. Since SCIs may present with cognitive or behavior impairment alone, young adults should be regularly screened for emerging declines. If positive, then a MRI to look for SCI would be appropriate. In addition, referral to a provider such as a psychologist who can provide more detailed assessment of cognitive function would be appropriate [64].

## Do Newer Medical Therapies Change Management of Arterial Ischemic Stroke, SCI, and MMS?

The emerging opportunities in gene therapy, which involves autologous transplantation of genetically modified hematopoietic stem cells, and more recently approved medications raise the question whether these might affect cerebrovascular disease and its management in children with SCA. However, this progress may come slowly. For instance, while a number of studies of gene therapy for sickle cell disease are actively recruiting in the United States, most exclude people with cerebrovascular disease [44]. Therefore, current trials may not provide information regarding the role of gene therapy for primary and secondary stroke prevention in SCA. Furthermore, multiple disease modifying medications have been approved in the last decade, but their role in the modification of cerebrovascular disease is still investigational. For instance, voxelotor stabilizes HbS in the oxygenated state to inhibit Hb polymerization and erythrocyte sickling [65]. Two active trials in children are examining the effect of voxelotor on transcranial Doppler velocities. (HOPE Kids; ClinicalTrials.gov NCT02850406), (HOPE Kids 2 study; ClinicalTrials.gov NCT04218084). In contrast, crizanlizumab (SelG1) downregulates endothelial expression of P-selectin, thereby reducing inflammation-mediated cell adhesion, vaso-occlusive episodes, and acute chest syndrome episodes in patients with sickle cell disease [66]. Cerebrovascular complications were not an endpoint of the initial trial, but a single site study is recruiting to examine crizanlizumab’s effect on SCI (Clinicaltrials.gov NCT05334576).

## Conclusions

A neurologist evaluating a child or young adult with SCA should have a heightened level of suspicion for the possibility of neurological complications. The patient presenting with an acute stroke or a clear TIA is diagnostically obvious, but we have presented several scenarios where one needs to consider SCA as an underlying diagnosis and/or contributor to a neurological presentation. This includes presentations that may not involve a typical neurologic finding but more subtle symptoms including deteriorating behavior, attention, or cognition. Furthermore, while children with SCA can develop common problems such as headaches and migraines just like other children, it is critical to recognize that these may also be related to cerebrovascular disease [67].

While the results of major clinical trials led to major advances in the prevention and management of SCA-related cerebrovascular disease in children there remain significant gaps in our knowledge that limit the long-term management of SCA, especially in adolescents and young adults. For example, what is the optimal management and treatment for young adults with SCA and stroke? How long and how frequently should one monitor these adults for the development of SCI? Also, how long and how frequently should patients with moyamoya vasculopathy be monitored, especially those with clinically asymptomatic aneurysms? One hopes that with time, the recently approved treatments for SCA provide opportunities for more effective modulation or suppression of cerebrovascular complications.

## Key References

- Reeves SL, Freed GL, Madden B, Wu M, Miller L, Cogan L, et al. Trends in quality of care among children with sickle cell anemia. Pediatr Blood Cancer. 2022;69(2):e29446. **The authors used Medicaid data from Michigan and New York to show that recommended antibiotic prophylaxis and TCD screening are not performed as recommended for most children with SCA. Their data show the gaps in care persist into the present era.**
- Davidow KA, Miller R, Phillips SM, Schlenz AM, Mueller M, Hulbert ML, et al. DISPLACE Study Shows Poor Quality of Transcranial Doppler Ultrasound for Stroke Risk Screening in Sickle Cell Anemia. Blood Adv. 2024;8(13):3444–52. **In this sub-analysis of the DISPLACE study the authors found significant variation in which vessels were assessed by transcranial Doppler, which velocities were used to define abnormal results, and in 52% of reports it was impossible to determine whether the reported TAMMV actually reflected what was measured.**
- Aldana PR, Hanel RA, Piatt J, Han SH, Bansal MM, Schultz C, et al. Cerebral revascularization surgery reduces cerebrovascular events in children with sickle cell disease and moyamoya syndrome: Results of the stroke in sickle cell revascularization surgery retrospective study. Pediatr Blood Cancer. 2023;70(7):e30336. **The results from this retrospective, multi-site study suggest that outcomes are better following cerebral revascularization surgery in children who have moyamoya syndrome in SCA. There is uncertainty whether revascularization surgery is necessary if children have chronic transfusion to reduce the HbSS burden.**
- Jones RS, Ford AL, Donahue MJ, Fellah S, Davis LT, Pruthi S, et al. Distribution of Silent Cerebral Infarcts in Adults With Sickle Cell Disease. Neurology. 2024;102(10):e209247. **This study showed that despite advances in SCA management and preventive treatment, nearly 50% of young adults in a 2 site study had multiple SCIs, with a median of 5.5 SCI per individual.**

## Data Availability

No datasets were generated or analysed during the current study.
